# The eTOX Data-Sharing Project to Advance *in Silico* Drug-Induced Toxicity Prediction

**DOI:** 10.3390/ijms151121136

**Published:** 2014-11-14

**Authors:** Montserrat Cases, Katharine Briggs, Thomas Steger-Hartmann, François Pognan, Philippe Marc, Thomas Kleinöder, Christof H. Schwab, Manuel Pastor, Jörg Wichard, Ferran Sanz

**Affiliations:** 1 Research Programme on Biomedical Informatics (GRIB), Hospital del Mar Medical Research Institute (IMIM), Department of Experimental and Health Sciences, Universitat Pompeu Fabra, C/Dr. Aiguader 88, Barcelona E-08003, Spain; E-Mails: mcases@imim.es (M.C.); manuel.pastor@upf.edu (M.P.); 2 Lhasa Limited, Granary Wharf House, 2 Canal Wharf, Leeds LS11 5PS, UK; E-Mail: katharine.briggs@lhasalimited.org; 3 Investigational Toxicology, Bayer HealthCare, Müllerstraße 178, Berlin D-13353, Germany; E-Mails: montserrat.cases@bayer.com (M.C.); thomas.steger-hartmann@bayer.com (T.S.-H.); joerg.wichard@bayer.com (J.W.); 4 PreClinical Safety, Novartis Institute for Biomedical Research, Klybeckstrasse 141, Basel CH-4057, Switzerland; E-Mails: francois.pognan@novartis.com (F.P.); philippe.marc@novartis.com (P.M.); 5 Molecular Networks GmbH, Medical Valley Center, Henke strasse 91, Erlangen 91052, Germany; E-Mails: kleinoeder@molecular-networks.com (T.K.); schwab@molecular-networks.com (C.H.S.)

**Keywords:** *in silico* toxicity, *in vitro* toxicity, *in vivo* toxicity, data sharing, data integration, ontologies, decision support system, predictive models, read-across, QSAR

## Abstract

The high-quality *in vivo* preclinical safety data produced by the pharmaceutical industry during drug development, which follows numerous strict guidelines, are mostly not available in the public domain. These safety data are sometimes published as a condensed summary for the few compounds that reach the market, but the majority of studies are never made public and are often difficult to access in an automated way, even sometimes within the owning company itself. It is evident from many academic and industrial examples, that useful data mining and model development requires large and representative data sets and careful curation of the collected data. In 2010, under the auspices of the Innovative Medicines Initiative, the eTOX project started with the objective of extracting and sharing preclinical study data from paper or pdf archives of toxicology departments of the 13 participating pharmaceutical companies and using such data for establishing a detailed, well-curated database, which could then serve as source for read-across approaches (early assessment of the potential toxicity of a drug candidate by comparison of similar structure and/or effects) and training of predictive models. The paper describes the efforts undertaken to allow effective data sharing intellectual property (IP) protection and set up of adequate controlled vocabularies) and to establish the database (currently with over 4000 studies contributed by the pharma companies corresponding to more than 1400 compounds). In addition, the status of predictive models building and some specific features of the eTOX predictive system (eTOXsys) are presented as decision support knowledge-based tools for drug development process at an early stage.

## 1. Introduction

Safety failure is one of the main reasons for attrition of new drugs, as reported recently in a retrospective review of Food and Drug Administration (FDA) documents between 2000 and 2012 [1]. Toxicity-related adverse events continue to be a leading cause of new drug candidates’ erosion at all stages, and there is a need for identifying predictable safety issues earlier during preclinical stages of the drug development process [2]. Despite predictive models being of increasing quality, the pharmaceutical industry still fail, for instance, predicting some cardiotoxicity events which are currently hard to detect until large Phase 3 trials [3]. Analysis of reasons for previous failures and exploitation of them should help in improving the efficiency of clinical development of new drugs and their safety profiles.

Several initiatives (ToxML [4], DSSTox [5], ACToR [6], ACuteTox [7], EPA’s ToxCast [8,9,10,11], Tox21 [12], OpenTox [13], ToxBank [14], ISSTOX [15], RepDose [16], Open PHACTS [17], Safe-T [18], and COSMOS [19]) are gathering chemical related toxicity information in a more unified way, which allows data mining and opens opportunities for model building [20,21,22], with the final aim of increasing the reliability of toxicity prediction, thus reducing experimental toxicological testing, improving the drug development attrition rate and obtaining safer chemicals and drugs.

The majority of preclinical data acquired during drug development are not available in the public domain due to project attrition, intellectual property issues and protection of competitive advantages. The European Federation of Pharmaceutical Industries and Associations (EFPIA) identified lack of data accessibility as a significant obstacle for the development of read-across approaches and other *in silico* tools to support risk assessment for new molecules entities (NMEs) and considered the European Union’s Innovative Medicines Initiative (IMI) as an appropriate framework for overcoming this difficulty. IMI [23,24] is a public-private partnership created to promote collaborative research projects focused on effective prediction of pharmacology and toxicology as critical aspects of the drug discovery process, as well as on knowledge management, education and training in the field.

The eTOX project, the full title of which is “Integrating bioinformatics and chemoinformatics approaches for the development of expert systems allowing the *in silico* prediction of toxicities” [25] is an IMI project to be carried out between 2010 and 2016 by a public-private partnering of 11 academic groups, six SMEs and 13 pharmaceutical companies. All these institutions work together to set up a software platform (eTOXsys), which includes an integrative database and a comprehensive collection of predictive models, with the aim of providing a new and additional tool for the design of new drug candidates on the basis of cumulative knowledge shared between the pharma companies that participate in the project [26]. Since the compilation and integration of chemical and toxicological data can contribute to improving knowledge, the eTOX consortium is collecting toxicology related data from both public and proprietary domains of small molecule compounds, excluding biologics and large molecules like oligonucleotides or peptides. The eTOX database is becoming one of the largest and most relevant preclinical databases for pharmaceuticals including access to historical information from systemic toxicity reports contributed by the EFPIA partners participating in the project. The collected data originate from systemic repeated dose studies of various durations (from sub-acute to chronic), carried out in rodent, dog and other non-rodent species. Related data on safety pharmacology, pharmacokinetics, pharmacodynamics, drug disposition, reprotoxicity and carcinogenicity studies are currently being captured as complementary information. There are also ongoing efforts for integrating data from previous toxicogenomics and toxicology initiatives (InnoMed PredTox [27], TG-GATEs [28] and DrugMatrix [29]); datasets extracted from the scientific literature (PubChem [30], ChEMBL [31,32]) and from regulatory agencies documents (EPARs [33]); and in the course of the project extension (ENSO grant), there are additional efforts to link existing information from clinical databases (*i.e.*, DailyMed [34], Drugbank [35]). In addition, the consortium screens literature and public domain resources in order to identify data, tools, methodologies or discussions that could be of use to eTOX. This curated and annotated information is compiled in the eTOXlibrary [36,37], and is open to public access.

This article describes the processes and protocols defined within the eTOX project to create the eTOX database, and how it has evolved through the project years, including a review of the difficulties overcome. Options for predictive models building and read-across analyses are presented, and the features of the integrated system developed (eTOXsys) are shown to illustrate its potential as a decision support knowledge-based tool for drug-induced toxicity assessment.

## 2. Results and Discussion

### 2.1. Legacy Data Gathering

One of the main building blocks for the eTOX database is the data from several thousands of repeated dose toxicity studies performed by the participating pharma companies, which are being integrated together with publicly available data sources (*i.e.*, RepDose [38], ChEMBL [31,32], EPARs [33]) and from biomedical literature by text mining. Up to the start of eTOX, early 2010, this proprietary information was locked up at each individual pharma company, inaccessible to anyone but the respective owners. eTOX enabled a data extraction process to make the data available in a machine readable format and to share it within the eTOX consortium in a way that protects the intellectual property and confidentiality of sensitive information: each company identified sensitivity classes for their data which subsequently determined whether the data could be contributed to eTOX, and whether data needed to be protected or could be freely shared. This process is regularly accompanied by continuous classification of new structures/data, especially in those companies where approval is mandatory for each report, even when the structure has been already shared in the eTOX database.

Depending on the data sensitivity (Table 1) the eTOX database contains four categories of data: Public (open access data including the EFPIA data that is made public upon request); Non-confidential (data, including chemical structure, shared within the project consortium); Confidential (toxicological data shared within the eTOX consortium, excluding structure and pharmacological information); and Private (used only internally in each company for model validation). Information classified as Public, Non-confidential and Confidential (the shared data part only) is accessible to all project participants; The not-shared part of the Confidential data is held by the “honest broker” of the project, Lhasa Limited, being accessible only by the original owner of the data, and by model developers within the consortium under secrecy agreements.

**Table 1:** Data sensitivity categories within the eTOX project.

Category	Access	Sharing	Usage
**Public**	Public upon request	Structure and all available data	Read-across analysis, Models building and validation
**Non-Confidential**	eTOX consortium	Structure and toxicological data	Read-across analysis, Models building and validation
**Confidential**	Honest broker and data owner	Toxicological data	Read-across analysis (without structure query), Models building and validation (without structure query)
**Private**	Data owner	None	Models validation

Briefly, the process of extraction and collection of data can be summarized as follows. Once the legacy reports overcome the company’s internal clearance steps, the data process continues within the EFPIA company, either extracting the data themselves or transferring the study report to a specialized Contract Research Organisation (CRO) for data extraction. Each individual EFPIA partner revises the CROs work, performing quality checks and then uploads separate files containing the non-confidential or confidential information to Lhasa Limited’s eTOX Vitic Nexus database server via a secure ftp connection. As soon as the data are received at Lhasa Limited, it is imported into an internal production database, or standalone database in the case of confidential information. Additional quality checks to confirm the agreed standards within the data entry guide and structure drawing guidelines are then performed. This ensures that the different partners enter the data in a consistent way and most common entry errors are detected. As data are added to the eTOX database, new verbatim terms are systematically identified and exposed into the curation tool developed within the project, the OntoBrowser (see hereafter). Curation to preferred terms is manually processed (when possible, standard terms are used to increase inter-operability, or at least terms are normalized to ensure global harmonization). Once the preferred terms have been assigned, these are incorporated into the database alongside the verbatim terms.

In order to track the data extraction process and evaluate its progressive advance, each EFPIA partner is required to provide information on monthly progress by submitting a “Reports tracking” worksheet that contains detailed information for every single report:

- Substance ID: The database substance identifier, created by the EFPIA partner;
- Report ID: The internal report name/identifier;
- Quality assessment: The result/status of the internal quality check;
- Clearance date: The timepoint when the report entered the eTOX extraction process;
- Status: Confidential/non-confidential;
- Progress: Sent to CRO date/Sent to Lhasa Limited date;
- Available at Vitic Nexus eTOX database: The date of the database release that contains the report;
- Progress comments: Comments when applicable;

Ending 2012, half of the EFPIA partners had contributed their first reports to the eTOX database (2012. 3 release contained 160 substances, 55 of them confidential, and 1073 study data records) and the CROs had completed the extraction of almost 75% of the cleared reports at that time (2087/2904). Progressively, data processing is gaining momentum: new batches of reports enter in this process and experience gained from the first reports helps in accelerating the whole cycle. The current release (2014. 2 launched in August 2014) includes data from 3046 legacy reports corresponding to 1403 substances (515 confidential, or 37%) and 4291 study data records.

### 2.2. Data Integration, Harmonization and Curation

To safeguard the intellectual property in the database, a trusted intermediary or honest broker, is required to host and maintain the database and manage data access permissions. Lhasa Limited, a private not-for-profit organisation with a proven track record in maintaining confidentiality of data, was selected for this role based on its reputation for secure data sharing [39].

To integrate and facilitate the sharing of all data extracted from legacy reports, Lhasa Limited created the eTOX database based on the previously developed Vitic Nexus software, a chemical relational database (CRD), which is chemocentric accompanied by fields containing data associated with that chemical. To cover storage of data coming from legacy reports, a comprehensive database schema has been developed in an iterative manner. The schema is self-describing and can easily be modified to meet the specific requirements. Currently, it includes different data blocks: chemistry data (*i.e.*, structure, identifiers, pharmacological action), study design information (*i.e.*, species, strain, sex, dosage, administration route), toxicokinetics (*i.e.*, dose, *T*_max_, *C*_max_), general toxic effects information (*i.e.*, dose, mortality, bodyweight), clinical chemical findings (*i.e.*, dose, clinical chemical parameter), clinical haematological findings (*i.e.*, dose, haematology parameter), gross necropsy (*i.e.*, dose, organ affected, pathology finding, number of animals affected), histopathological findings (*i.e.*, dose, organ affected, histopathology finding, number of animals affected), and ADME (absorption, distribution, CYP450 identification, metabolite identification, excretion balance and clearance information).

One of the key requirements of the eTOX database is to implement procedures guaranteeing a consistent representation of the compound chemical structures. Only then can records representing the same compound from different sources be recognized as such. This issue is not trivial and required setting up detailed guidelines for transcribing the structures into the database in a consistent way (e.g., how to introduce salts, tautomers, racemic mixtures, * etc.*), which enforces its application. Compounds from external sources are introduced using the same guidelines. Structure matching is identified using standard identifiers (CAS registry numbers) or canonical representations of the compound structure (InChi) [40].

To optimize the utilization of the contributed data, the eTOX database needed a unification of the diverse terms extracted from different sources. The eTOX data-sharing exercise responds to what was claimed by Harland *et al.* in 2011 [41]: That there is a need of empowering industrial research with shared biomedical vocabularies, which is essential for the further exploitation of the continuous data generation and storage.

Ongoing work to develop a common ontology is progressing among the eTOX consortium. The goal is to reuse what is available in order to increase inter-operability with other databases, but also to enhance or even create *de novo* code lists and ontologies that are needed. One of the main challenges for toxicology is the ongoing implementation of the Standard for Exchange of Nonclinical Data (SEND) [42] by the Clinical Data Interchange Standards Consortium (CDISC) [43]. This is an application of the CDISC Standard Data Tabulation Model (SDTM) as a way to present non-clinical data in a consistent format. It is also a key driver for standardization of code lists describing experimental designs and consolidation of terms used to describe key toxicology results. eTOX is aiming at aligning its terminologies to SEND to make them compatible.

One of the areas where standardization is critically needed for eTOX is that of histological pathology findings. It is widely known as a very challenging task since observations are currently not standardized and observations can be reported using various terms. Pathologists assembled to tackle the task and work is in progress. The most advanced efforts are the International Harmonization of Nomenclature and Diagnostic Criteria (INHAND) (former reference for nomenclature and diagnostic criteria in toxicologic pathology RENI) [44] related terminology, used as a standard by current SEND draft. While these efforts are under way, eTOX invested massively into it with two goals: (1) Refine code lists while trying to maintain compatibility; and (2) Create a tree of terms in order to facilitate later search. Novartis is leading this task as the company was already working on glossaries mapping applications and ontologies for pre-clinical findings before the eTOX project started, and therefore was more advanced in this area than most of the other EFPIA partners. It is worth noting that members of eTOX consortium are involved in both SEND and INHAND subteams, linking the two efforts.

**Figure 1:**
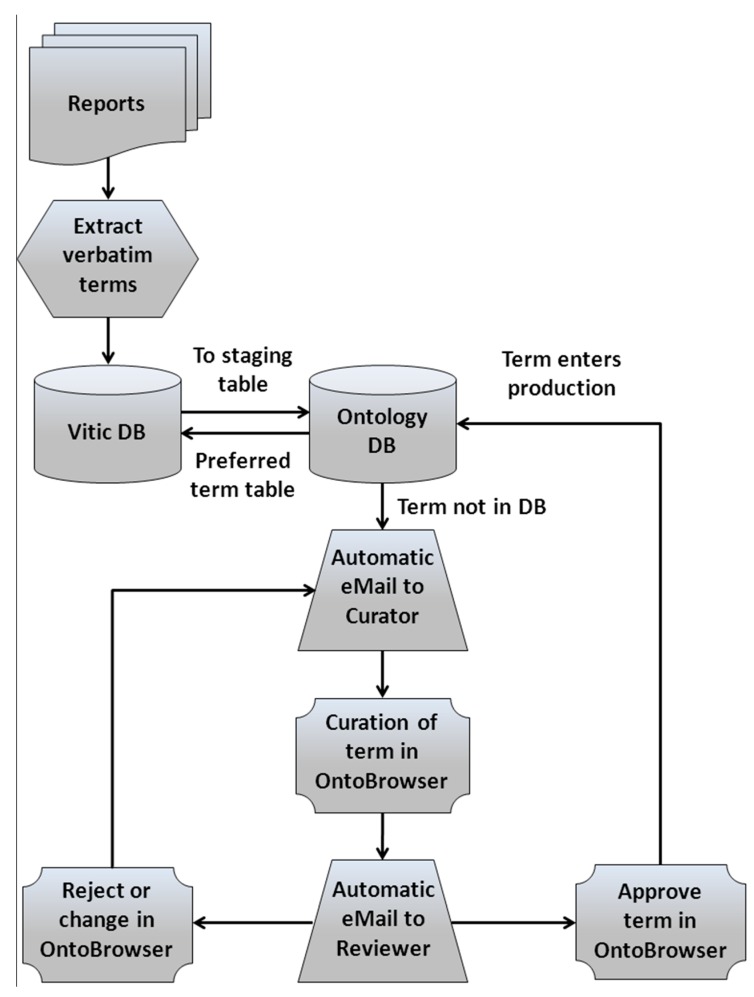
The process for verbatim terms curation and communication between Vitic Nexus eTOX database and the OntoBrowser database.

A major area of work for eTOX is the curation of verbatim terms to standard terms used by the consortium (Figure 1). An ontology curation core team is in charge of populating the different ontology modules with verbatim terms recorded in the database as synonyms of the stated assigned preferred terms. It is up to curators to determine whether a term is assigned to a previously defined term or if there is a need to open a new entry. In order to handle the code list and ontologies generation/mapping/curation by toxicology experts, Novartis developed a web-based ontology curation tool called OntoBrowser (to be released as open source) that allows the curation of the terms extracted from the legacy reports through a front-end interactive graphical interface with search and curation functions. New verbatim terms added to the eTOX database are passed to the OntoBrowser tool where the curation team can then map them to the ontologies. Always, a second scientist with approval privileges checks and confirms each specific mapping. The resulting preferred terms are then integrated back into the eTOX database so users can see both: verbatim and preferred terms alongside each other. So far, more than 8 million verbatim terms were assigned to preferred terms, representing 97% of the total number of terms in the standardized fields of the current release of the eTOX database. That large and complex effort is a guarantee to obtain high quality and usable data.

Leveraging the eTOX experience to create guidelines and tools for data curation, the eTOX consortium is working with standards bodies to promote the eTOX ontologies as standards, including making them compatible with SEND. In this regard, currently part of the eTOX database contents have been harmonized with the SEND controlled vocabularies loaded onto the OntoBrowser tool.

The mapping for harmonization and the curation work are essential for allowing high-quality read-across analyses of the eTOX database contents. The common ontology will become a key deliverable of the eTOX project because it will contribute an industry standard ontology for pre-clinical findings that is sorely lacking in this field. These common efforts will build confidence and consistency in the quality of the data shared, and will help in generating realistic statistics for its contents, apply read-across approaches and develop quantitative structure-activity relationship (QSAR)-like or other types of predictive models. For details, see reference Briggs *et al.* [45] for a comprehensive statistical analysis of the Vitic Nexus eTOX database 2014. 1 release contents, and Hewitt *et al.* [46] article as an example of challenges for read-across analysis application.

### 2.3. Data Analysis for Modeling Purposes

As aforementioned, the main uses planned for the eTOX database are data browsing, read-across analysis and building of computational models to predict drug-induced toxicity. The CRD design of the database permits its exploitation across both chemical (exact structure, substructure and structural similarity searches) and toxicity-related biological domains (searches for specific findings or study design parameters).

However, it must be emphasized that the shared toxicological studies were originally not designed for generating predictive models. The eTOX database essentially contains a collection of “findings”, observations of a certain biological endpoint after the administration of a compound at given dose, in one or more animals, for a given time. The use of this data for model building needs to address three inconvenient aspects: (1) The use of this information for attributing the compounds to some generic biological property requires a previous inference operation that considers the circumstance of the observation: Was it observed at a very high dose? Was it present for a relevant number of animals? Was the effect significantly different from those observed for controls? Was the animal moribund?; (2) Modeling requires that the compound properties were consistently assigned, which means that properties observed at different doses, in diverse species or using diverse administration routes cannot be compared directly; and (3) Most observable phenotypes (e.g., cholestasis) represent the manifestation of multiple toxic mechanisms that cannot be easily covered by a single model.

**Figure 2:**
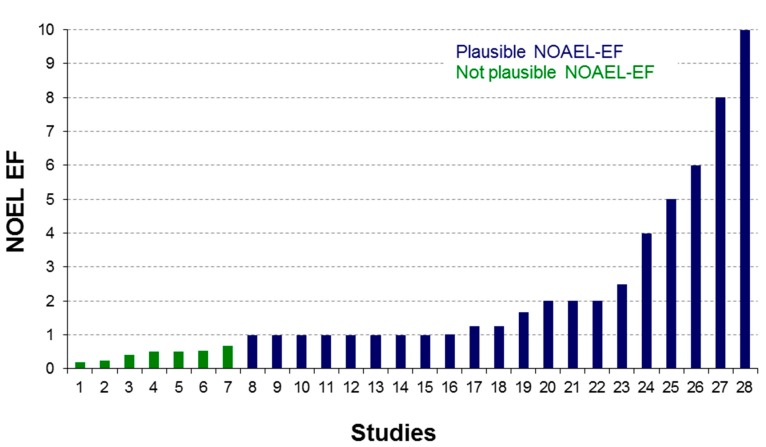
Preliminary derivation of an extrapolation factor (EF) for NOAEL from 2–4-week studies from the eTOX database.

The eTOX database can be used to analyze relationships between study duration and species-specificity of toxic effects. As an example, the database was searched for compounds for which a “no observed adverse effect level” (NOAEL) has been determined in studies of various durations. The ratio of studies with NOAEL value between long-term and short-term has been calculated and the distribution analyzed. Although the number of studies, for which NOAELs were explicitly provided for different study durations is low, a first analysis was performed for 28 studies from a single company comparing the NOAELs from 2-week toxicity studies to those of 4-week toxicity studies (see Figure 2). Although the data set is still too small to come to a generalized conclusion, the analysis points towards a median extrapolation factor in the range of 2. A few compounds were identified for which the factor was below 1, which is considered to be improbable, because it would indicate a lower toxicity during longer administration periods. The in-depth analysis of the results is still ongoing, but a potential reason could be induced metabolism of the compound during prolonged administration periods.

### 2.4. eTOXsys: The Data Browser and the Predictive System

**Figure 3:**
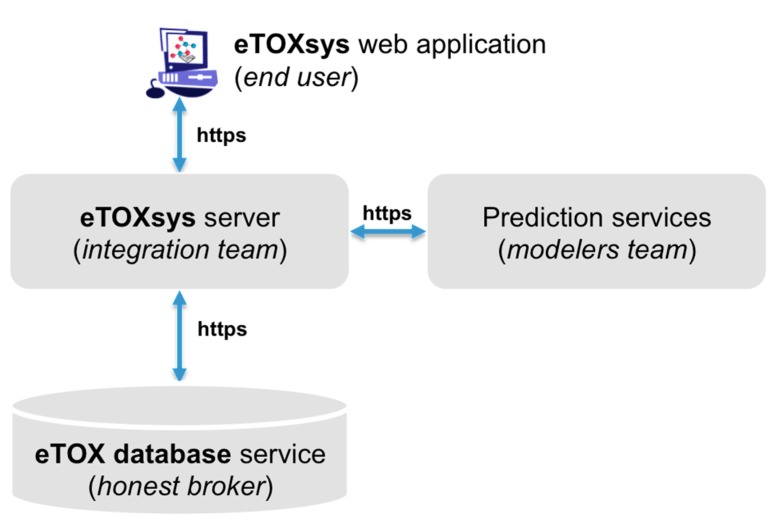
Scheme of the eTOXsys architecture.

The eTOXsys is a unified software platform integrating the various tools, databases and results achieved during the course of the project. It has been developed by Molecular Networks GmbH. This integrated software system provides access to the predictive models and databases through a uniform user interface to support the hazard identification and risk assessment of drug candidates queried into the system. The system architecture of eTOXsys platform was designed within the consortium during the first two years of the project. The eTOXsys is a web-based system and can be accessed by the end user through a standard web browser. The architecture comprises a decoupled and distributed system in order to take into account the various technologies and methods used by the contributing service providers (databases and predictive models). The services are hosted, provided and maintained by the individual contributing partners who developed the model. The system consists of four main components (Figure 3). The eTOXsys web application facilitates the access to the various services and is provided by the central eTOXsys server to the end user. Furthermore, the eTOXsys server marshals user requests for toxicological endpoint predictions (provided by the prediction services) and database searches (provided by the eTOX database service), collects the results from the corresponding web services and returns them to the end user web application.

The web application, *i.e.*, the eTOXsys user interface, has been designed and implemented in an intuitive and user-friendly manner. Chemical compounds can be input by names, registry numbers, sketched by an interactive molecule editor, or by uploading chemical files (such as Molfile or SMILES). The database search and prediction results are returned to the web application and presented to the end user in a structured layout as interactive hit lists, study design, dose range and effects tables (for database searches) and prediction results pages (for prediction services). The database query and prediction results can be further refined and analysed by the end user and finally exported in chemical and worksheet-compatible standard file formats (such as SD or Excel files).

As previously indicated, the eTOXsys offers two major use cases, which are retrieving toxicity information for the molecules stored in the eTOX database, and predicting toxicity endpoints for molecules for which no appropriate information is available in the database.

**Figure 4:**
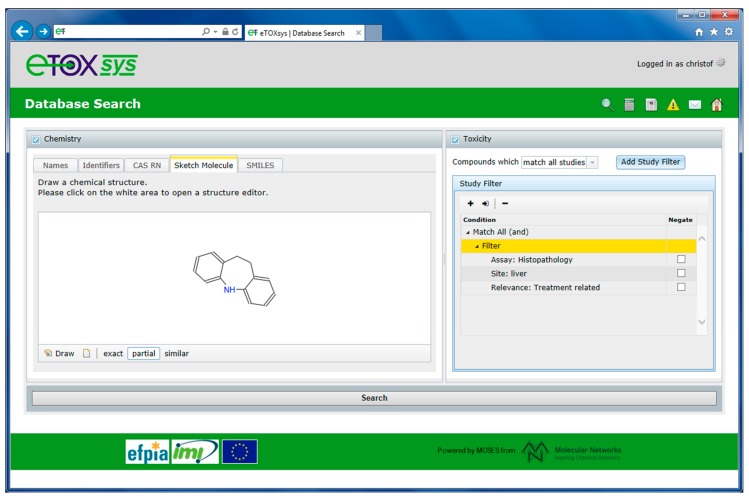
Combined chemistry and toxicity database search.

For database queries, the eTOXsys interface allows for performing chemistry-based searches and toxicity-based searches. For chemistry searches, the database can be queried for chemical structures including name, registry number, full-structure, substructure and structural similarity. For toxicity searches, scientific hypothesis-driven (single or multi parameter) queries can be formulated through an interactive query builder that has been designed in close collaboration with the toxicologists. Toxicological-related information can be searched by study designs, such as species, route of exposure and duration, as well as by assay type, target organ and site, effect level, finding and treatment-related relevance. Queries can be built up by defining multiple criteria and linking them using Boolean logic. In addition, the query builder for formulating the toxicity searches makes use of the eTOX ontologies as users can define their searches using the preferred terms assigned, if already available. Furthermore, chemistry- and toxicity-based searches can be performed in combination focusing on a particular chemical space (or compound class) and specific toxicological effects. Figure 4 illustrates such a combined chemistry- and toxicity-based search (hypothesis-driven search). The database is searched for all compounds that exhibit the benzazepine moiety depicted on the left side of the query form (substructure search). In addition, the retrieved compounds should show histopathology findings (assay type) in liver (target site) that are treatment related (relevance).

**Figure 5:**
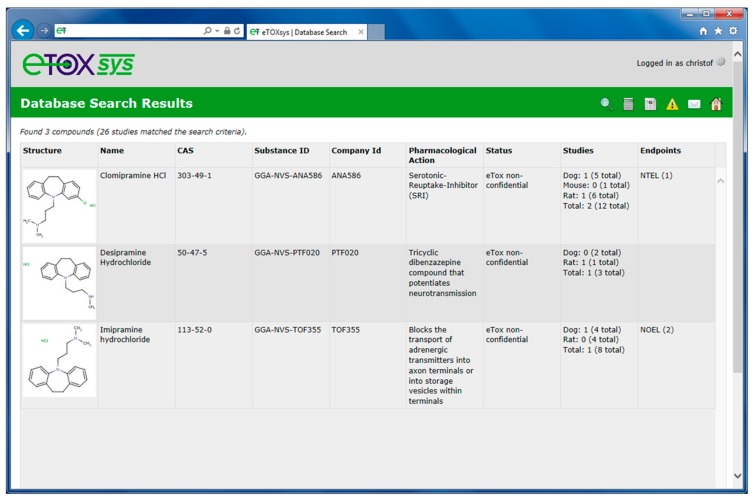
Hit list from a combined chemistry and toxicity database search.

Figure 5 shows the hit list of three compounds that were retrieved from the database and that match the combined chemistry and toxicity search criteria displayed in Figure 4. In the hit lists, the retrieved chemical structures are shown along with their names, registry numbers and IDs, the pharmacological action and the number of available studies in the various species. Numerical endpoints, if available, are displayed as well.

**Figure 6:**
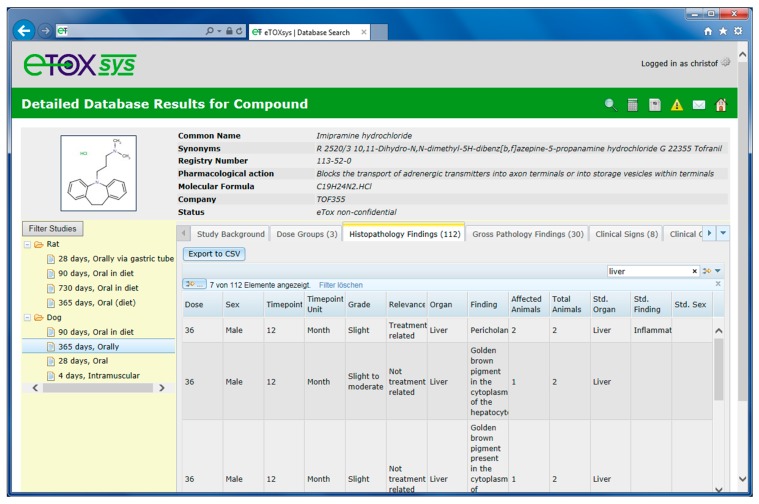
Detail page of a molecule displaying the toxicity data stored in study design and effects tables.

The rows in the hit lists are linked to the detailed toxicity information pages of each compound. Figure 6 shows the detailed page for the compound imipramine hydrochloride (third in the hit list in Figure 5) and the treatment related effects found in liver in a 365 days dog study with oral route of exposure.

In order to improve decision making in drug discovery and ensure the benefit of the data sharing and integration implemented by the eTOX project, some of the common decisions that toxicologists and medicinal chemists face in their daily jobs were collected from EFPIA partners to list potential hypothesis-driven (single or multi parameter) queries to the database contents.

The second major use case of the eTOXsys is the prediction of toxicological relevant properties of chemical compounds applying the *in silico* models developed in the framework of the project [47,48,49,50,51,52,53]. Although, as the current version does not yet include models making use of the legacy report data contributed, for the aforementioned reasons, strategies for utilising the *in vivo* data have been refined, and the modeling technology used by eTOXsys has been developed for a large number of toxicologically relevant endpoints using public data. At present the eTOXsys online version includes 74 models covering diverse endpoints: 28 ADME, five transporters, two physicochemical properties, two carcinogenicity, two genotoxicity, 16 organ toxicity, and 19 safety pharmacology, which were developed as QSAR and non-QSAR-like models

**Figure 7:**
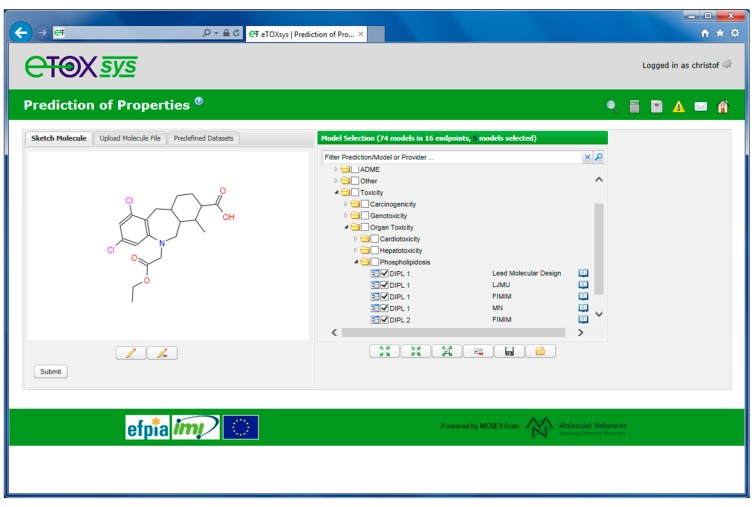
Tree representation of available prediction models for the Phospholipidosis prediction endpoint.

Figure 7 shows the user interface of eTOXsys for the request of toxicity-related predictions. Chemical structures can be entered by sketching a molecule or by uploading a chemical file (such as a Molfile or a SMILES file). The available models are organized in a tree format applying a hierarchy that has been developed in collaboration with the EFPIA toxicologists. This hierarchy groups the predictive models into categories of related endpoints, e.g., the prediction models for drug-induced phospholipidosis are grouped into the hierarchy “Toxicity” → “Organ Toxicity” → “Phospholipidosis”.

**Figure 8:**
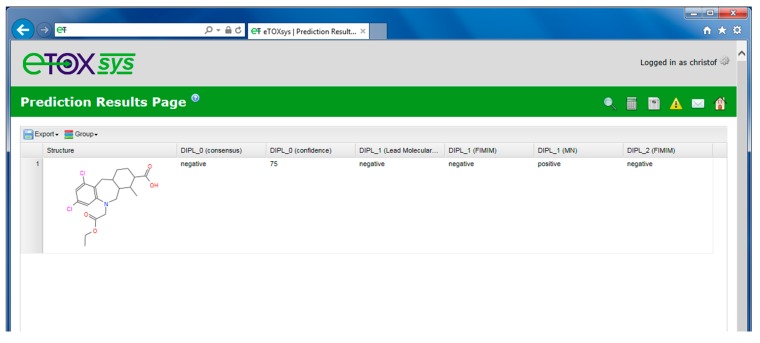
Prediction result table (including a consensus outcome).

The results of a performed prediction are presented to the user in a table view, including the molecular structure(s) and the predictions obtained for the requested models. If more than one prediction model is available for a specific endpoint and they are selected by the user, an automatic consensus prediction is provided. The consensus prediction is based on a qualitative weighting (“majority vote”) of the predictions of the individual models. In addition, a confidence score is provided based on the rate of votes of the individual models (see Figure 8, columns 2 and 3 next to the structure depiction). When no majority is reached for an outcome, the consensus prediction returns the value “unknown” and the confidence is set to “NA”.

**Figure 9:**
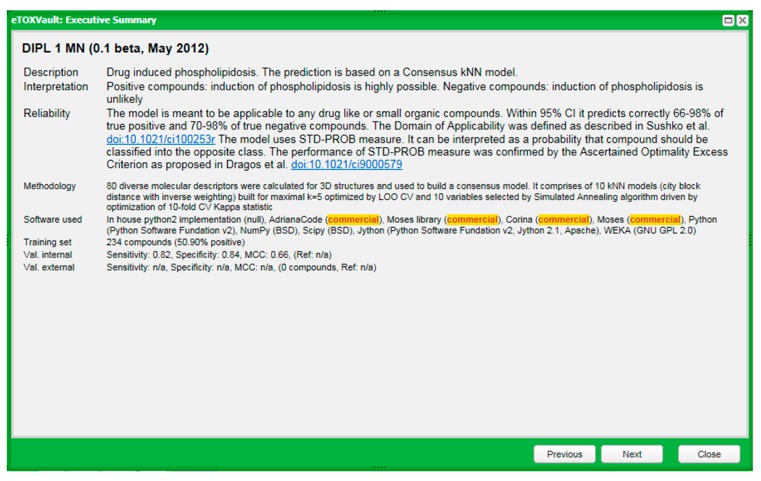
Example of an Executive Summary of prediction model characteristics.

The key requirement of the eTOX project is that all the models have to be appropriately documented, according to well-accepted standards. With this aim, a centralized relational database and a web interface called eTOXvault has been created by Fundació IMIM (FIMIM), one of the eTOX partners, in order to store data and methodology used to build each model. It can be accessed by the end users at different levels of detail, from a fiche called Executive Summary that provides support for interactively selecting the models and interpreting the model results (see an example for one Phospholipidosis model in Figure 9) to a more extended dossier providing exhaustive information about the methodologies used.

An added valuable feature of eTOX predictive models is that the predictions are always accompanied by metrics indicating how reliable they are. Some QSAR models developed in the framework of eTOX make use of ADAN, a new method developed *ad hoc* to provide a robust assessment of the model applicability domain [54].

During the development phase, an on-line version of the eTOXsys is accessible consortium-wide, even if in the final version, and the whole system will be deployed at the participant intranets (EFPIA partners), thus guaranteeing the full confidentiality of queries and predictions and allowing inclusion of private confidential data. In order to deploy such a complex system consisting of various web service-based components and taking into account the different IT infrastructures and environments at the EFPIA partners, the use of virtualization techniques (or “virtual machines”) was pursued. A proof-of-concept (PoC) study of this strategy was carried out with a reduced set of functionality of the eTOXsys in order to identify potential pitfalls or bottlenecks for the deployment process. After the PoC deployment exercise was successfully finalized, a set of standard operating procedures, communication channels, guidelines and configuration tools (including a web-based knowledge base to support IT personnel) has been established to improve and speed up the deployment process. The deployment of the eTOXsys version 2 is currently ongoing.

**Figure 10:**
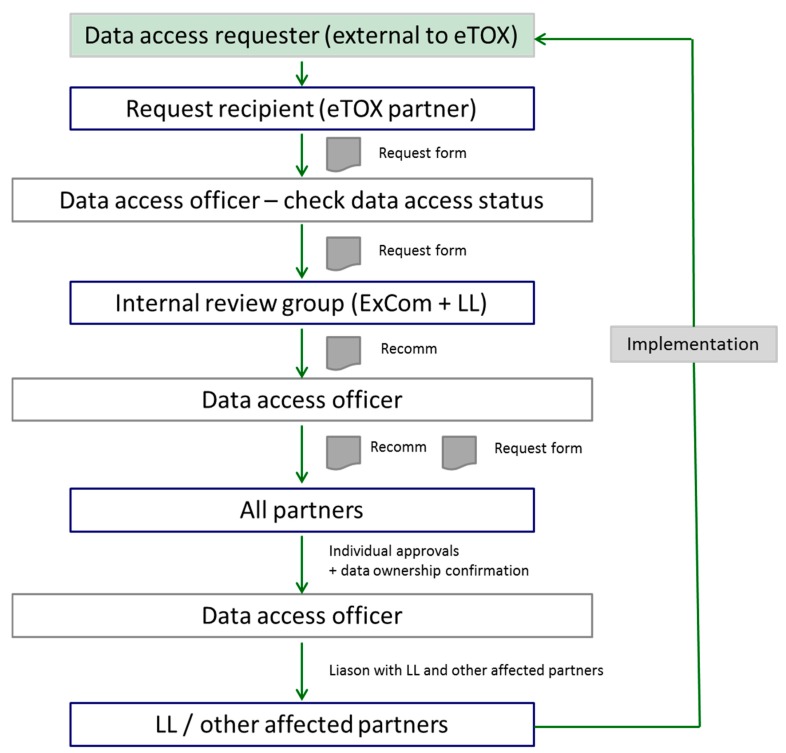
Data access strategy workflow (LL is the abbreviation of Lhasa Ltd.).

### 2.5. Ongoing and Future Actions

The eTOX project started early 2010, and will last till ending 2016. This upcoming period will serve to continue filling the eTOX database with new contributions from the EFPIA partners and new efforts of gathering toxicological related data from the public resources. Exploration for novel strategies for developing multi-scale and multilevel models will be addressed based on the compendious of data available. Since the eTOX database is becoming one of the largest and most relevant pre-clinical databases for pharmaceuticals including access to historical information from systemic toxicity reports, the project has already received several requests from third parties for access, e.g., to validate and assess adverse outcome pathways (AOP) that are being developed. At present, access to the eTOX database is only available to the members of the eTOX project. To accommodate such external data requests, the project has developed a workflow, which is depicted in Figure 10 and briefly can be narrated as follows: the external requesters contact an eTOX partner or the eTOX Executive Committee (ExCom), and Lhasa Limited (LL), describing his request and the desired data; this request is transferred to the eTOX data access officer, who analyzes the confidentiality status of the requested data sets; subsequently, an internal scientific review resulting in a recommendation (Recomm) is performed, and the result of this recommendation (yes/no) is forwarded to the data access officer who then informs the data owners accordingly; eventually, the final decision lies in the hand of the data owner, who then either performs the query or transfers the requested data to the third party.

The eTOX consortium is committed to collaborate with other IMI projects and related international initiatives, to outreach the health authorities and regulatory agencies for eTOXsys usage, and to facilitate progressively access to the eTOX results by the broader scientific community. The eTOX project will evolve with the expectation of enforcing new scientific advances and knowledge creation in the field of the global drug discovery from the side of the *in silico* toxicity prediction. Moreover, we are convinced that data sharing will become the norm in pharmaceutical R&D, propelled by electronic submission and transparency requirements, and eTOX is paving that way in this respect.

## 3. Conclusions

Within the frame of the IMI-eTOX project a large database with in vivo legacy toxicity data from more than 4000 studies was developed. Platforms for multi-parametric searches and predictive models were created. It is expected, that this project will set new standards in the field of data sharing, curation and modelling.
